# Impairments in Brain Bioenergetics in Aging and Tau Pathology: A Chicken and Egg Situation?

**DOI:** 10.3390/cells10102531

**Published:** 2021-09-24

**Authors:** Amandine Grimm

**Affiliations:** 1 Transfaculty Research Platform Molecular and Cognitive Neuroscience, University of Basel, 4002 Basel, Switzerland; amandine.grimm@unibas.ch; 2 Neurobiology Lab for Brain Aging and Mental Health, Psychiatric University Clinics, 4002 Basel, Switzerland; 3 Life Sciences Training Facility, University of Basel, 4055 Basel, Switzerland

**Keywords:** tau protein, bioenergetics, brain glucose metabolism, mitochondria, tauopathy, Alzheimer’s disease, lifestyle factors, diet, exercise, cognitive stimulation

## Abstract

The brain is the most energy-consuming organ of the body and impairments in brain energy metabolism will affect neuronal functionality and viability. Brain aging is marked by defects in energetic metabolism. Abnormal tau protein is a hallmark of tauopathies, including Alzheimer’s disease (AD). Pathological tau was shown to induce bioenergetic impairments by affecting mitochondrial function. Although it is now clear that mutations in the tau-coding gene lead to tau pathology, the causes of abnormal tau phosphorylation and aggregation in non-familial tauopathies, such as sporadic AD, remain elusive. Strikingly, both tau pathology and brain hypometabolism correlate with cognitive impairments in AD. The aim of this review is to discuss the link between age-related decrease in brain metabolism and tau pathology. In particular, the following points will be discussed: (i) the common bioenergetic features observed during brain aging and tauopathies; (ii) how age-related bioenergetic defects affect tau pathology; (iii) the influence of lifestyle factors known to modulate brain bioenergetics on tau pathology. The findings compiled here suggest that age-related bioenergetic defects may trigger abnormal tau phosphorylation/aggregation and cognitive impairments after passing a pathological threshold. Understanding the effects of aging on brain metabolism may therefore help to identify disease-modifying strategies against tau-induced neurodegeneration.

## 1. Introduction

Aging is the main risk factor for a wide range of diseases, especially neurodegenerative disorders. Brain aging is marked, among others, by bioenergetic defects and increase in oxidative damages [1]. The brain is the most energy-demanding organ in the body. Despite its relatively small size (2% of the total body weight), the brain accounts for 20% of the energy expenditure for an individual at rest [2]. Neurons are the brain cells that have the highest energy demand (70–80% of total energy), compared to glial cells (astrocytes, microglia, and oligodendrocytes) that share the remaining portion. This energy is mainly used to re-establish the ion balance after the firing of an action potential, to lower intracellular calcium levels, energize vesicle transmitter uptake, and allow mitochondrial transport by motor proteins [3]. Therefore, defects in brain energy metabolism will ultimately lead to cellular impairments, ranging from subtle alterations in neuronal function to neurodegeneration.

Tau is a protein belonging to the microtubule-associated proteins (MAPs) family, which stabilizes the assembly and function of microtubules [4]. In neurons, tau plays a role in axonal transport, cell polarity, and neurotransmission. Alterations in the properties of tau, such as aberrant hyperphosphorylation leading to tau aggregation and assembly into neurofibrillary tangles (NFTs), is characteristic of tauopathies, including Alzheimer’s disease (AD), progressive supranuclear palsy (PSP) and corticobasal degeneration (CBD). 

Abnormal tau hyperphosphorylation reduces its binding affinity to microtubules, therefore reducing tau microtubule-stabilizing function, which causes microtubule depolymerization [5,6]. Tauopathies can be separated into two categories:

(i) Primary tauopathies, such as PSP and CBD but also frontotemporal lobar degeneration (FTLD), in which tau plays a central role and mutant forms of tau have been identified [4].
(ii) Secondary tauopathies, such as AD, in which tau also plays a pathological role but other major factors appear to be involved (e.g., amyloid-β accumulation in AD). Although abnormal tau protein is clearly a hallmark of AD, no mutation in the tau-coding gene has been so far linked to the disease, suggesting that other factors are responsible for abnormal tau hyperphosphorylation and aggregation.

Even though substantial evidence has linked abnormal tau to neurodegeneration, the mechanisms underlying tau-induced neuronal dysfunction and death are still incompletely understood. 

Similar to what is observed during brain aging, numerous studies have demonstrated that abnormal tau protein induces bioenergetic defects and increases oxidative stress in the brain [7]. In particular, abnormal tau has a direct impact on mitochondria, the energy-producing organelles in the cells, leading to bioenergetic impairments and alterations in neuronal function. Strikingly, both tau pathology and brain bioenergetic defects seem to correlate with the clinical progression of AD, namely cognitive impairments, which does not appear to be the case for amyloid-β pathology (see Section 3.2. Abnormal tau and Brain Glucose Metabolism).

Therefore, the aim of this review is to highlight the links between abnormal tau and brain bioenergetic impairments, independently of amyloid-β. First, impacts of aging and tau pathology on brain bioenergetics will be summarized. Then, evidence showing that bioenergetic defects *per se* can trigger tau pathology will be discussed. Finally, the influence of lifestyle factors (diet, physical exercise, cognitive stimulation), known to have beneficial effects on brain metabolism and cognition, on tau pathology will be examined. 

## 2. Brain Bioenergetics and Aging

**Figure 1:**
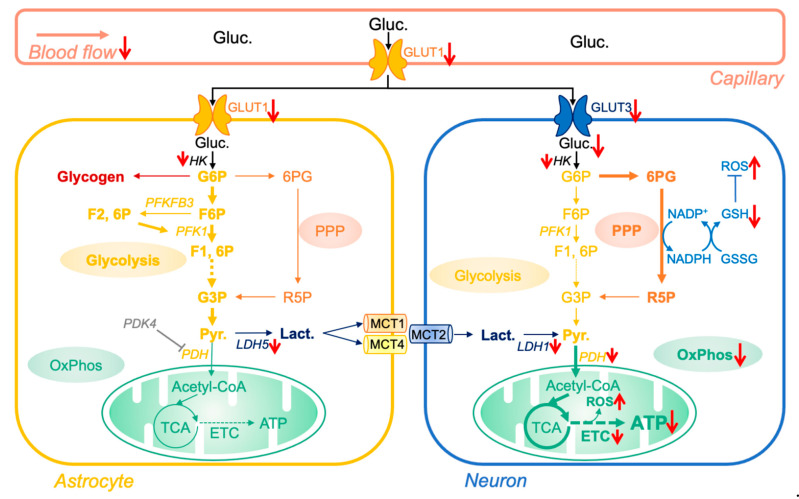
Impact of aging on brain glucose metabolism. Glucose (Gluc.), derived from nutritional sources, is transported to the brain through the blood flow. After entering astrocytes via glucose transporter 1 (GLUT1), glucose is either metabolized via the glycolytic pathway to form pyruvate (Pyr.) or stored as glycogen. Pyruvate is then converted into lactate by the lactate dehydrogenase 5 (LDH5) and, according to the astrocyte-neuron lactate shuttle hypothesis, transported to neurons via the monocarboxylic acid transporters 1, 2, and 4 (MCT1 and 4 at the astrocyte membrane, MCT2 at the neuron membrane). Due to the pyruvate dehydrogenase kinase 4 (PDK4)-dependent inhibition of pyruvate dehydrogenase (PDH), astrocytes have only limited oxidative phosphorylation (OxPhos) activity and rely on glycolysis for ATP production. Lactate entering in neurons is converted to pyruvate via the lactate dehydrogenase 1 (LDH1) and enters in the tricyclic acid cycle (TCA), generating substrates for OxPhos and ATP production. Glucose can also enter neurons via the glucose transporter 3 (GLUT3). However, due to the constant proteasome degradation of the 6-phosphofructo-2-kinase/fructose 2,6-biphosphatase, isoform 3 (PFKFB3), neurons have low glycolytic capacity. Therefore, glucose is mainly metabolized via the pentose phosphate pathway (PPP) to generate reduced nicotinamide adenine dinucleotide phosphate (NADPH). NADPH is then used to produce oxidized antioxidants such as glutathione, which are paramount to regulate the reduction/oxidation state in neurons. Of note, reactive oxygen species (ROS) are by-products of OxPhos activity, which are neutralized by antioxidant defenses (e.g., GSH/GSSG system). The red arrows indicate proteins and molecules that are impacted (*↑* increased/*↓* decreased) in brain aging. Abbreviations: 6PG, 6-phos-phogluconate; ETC, electron transport chain; F6P, fructose-6-phosphate; F1, 6P, fructose-1,6-diphosphate; F2, 6P, fructose-2,6-diphosphate; G3P, glyceraldehyde-3-phosphate; GSH/GSSG, reduced/oxidized glutathione; HK, hexokinaseG6P, glucose-6-phosphate; G3P, glucose-3-phosphate; PKF1, phosphofructokinase 1; R5P, ribulose-5-phosphate.

Glucose is the main source of energy in the brain. Molecules of glucose are metabolized via two main energetic pathways to produce adenosine triphosphate molecules (ATP): cellular glycolysis (generates 2 molecules of ATP) and mitochondrial oxidative phosphorylation (OxPhos, generates 36 molecules of ATP). Glucose, derived from nutritional sources, is transported through the blood flow to the brain capillaries. It enters the brain via glucose transporter 1 (GLUT1, 55-kD isoform) expressed in the endothelial cells of the blood-brain-barrier (BBB) [2] (Figure 1). Then, glucose is delivered to glial cells, namely astrocytes, via GLUT1 (45-kD isoform) and to neurons via GLUT3. Neurons have very low glycolytic capacity due to the constant proteasome degradation of the master regulator glycolytic enzyme 6-phosphofructo-2-kinase/fructose 2,6-biphosphatase, isoform 3 (PFKFB3). Therefore, they rely almost exclusively on OxPhos for ATP generation. Nevertheless, neurons can metabolize glucose through the pentose phosphate pathway (PPP), which allows the generation of reduced nicotinamide adenine dinucleotide phosphate (NADPH). NADPH molecules play an important role in neurons as they act as cofactor to generate reduced glutathione (GSH), major component of cellular antioxidant defenses. 

In contrast, astrocytes possess a high glycolytic capacity and generate lactate via conversion of pyruvate by lactate dehydrogenase 5 (LDH5) (Figure 1). Of note, astrocytes also have the possibility to store energy by converting glucose in glycogen, which constitutes the largest energy reserve in the brain [2]. According to the astrocyte-neuron lactate shuttle hypothesis, astrocytes take up glucose from cerebral capillaries and produce lactate which is released into the extracellular space [8]. Extracellular lactate is then taken up by neurons and converted to pyruvate by lactate dehydrogenase (LDH1), which enters the TCA cycle followed by OxPhos in neuronal mitochondria to generate ATP (Figure 1). 

Today, it is well accepted that brain aging is marked by a decrease in glucose use, an increase in mitochondrial dysfunction and oxidative damages that may alter neuronal integrity after passing a certain threshold. As these topics have already been reviewed in detail elsewhere [1,2], we will only briefly summarize key points regarding these important age-related phenomena.

### 2.1. Alteration in Glucose Metabolism

Normal aging is characterized by a progressive glucose hypometabolism in the brain. This phenomenon is further exacerbated in neurodegenerative disorders, including Alzheimer’s disease (AD) [2,9]. Indeed, it appears that with increasing age, the brain loses the ability to transport/metabolize glucose, which may cause age-related cognitive impairment due to insufficient brain energy supply [2,10]. Goyal and colleagues showed that the fall in brain glucose metabolism with aging is largely due to the loss of glycolytic activity in the brain [11]. Several causative factors can be proposed (Figure 1):

(i) Aging is associated with a decrease in cerebral blood flow, as well a loss of BBB integrity [12,13,14]. This may limit nutrient, including glucose, import into the brain.
(ii) Age-related reduction in the expression of glucose transporters as well as expression and/or activity of enzymes involved in glycolysis were reported (Figure 1) [15,16,17].

Combined, these clearly identified, yet unexplained, phenomena contribute to glucose hypometabolism in the aging brain.

### 2.2. Alteration in Mitochondrial Function

Often depicted as the powerhouses of the cell, mitochondria are not only the main generators of ATP molecules via OxPhos, but they are also involved in other processes such as calcium homeostasis and apoptosis. As neurons rely almost exclusively on OxPhos to generate ATP, defects in mitochondrial function will ultimately lead to cellular impairments, ranging from subtle alterations in neuronal function to neurodegeneration. 

Mounting evidence highlights mitochondrial impairments in the brain with increasing age (reviewed in [1]). Such age-related impairments include:

(i) Decreased mitochondrial bioenergetics, namely a decrease in the expression/activity of mitochondrial complexes (including complex I, III, IV and V) involved in OxPhos, decreased mitochondrial respiration (OxPhos) and ATP production.
(ii) Defects in mitochondrial dynamics (fusion/fission activity), mitochondrial transport/distribution, and mitophagy. Indeed, mitochondria are very dynamic organelles that constantly fuse and divide to mix their protein, lipid and mitochondrial DNA (mtDNA) content. Therefore, a decrease in mitochondrial quality control (mitochondrial dynamics/mitophagy) would lead to an accumulation of damaged mitochondria, and accumulation of mtDNA mutations. mtDNA encodes subunits of complexes of the electron transport chain (ETC, complex I, III, IV). Evidence shows that the accumulation of mtDNA mutations over time is a central mechanism driving aging and age-related diseases, as they may lead to decreased ETC activity and impaired mitochondrial respiration [1,18].

### 2.3. Increase of Oxidative Damages

Mitochondria are not only the main ATP generators, but they are also the main source of harmful reactive oxygen species (ROS) derived from OxPhos activity. In 1956, Harman enounced the “free-radicals theory of aging”, which postulates that aging, as well as age-associated degenerative diseases, is a consequence of free-radical attacks on cells and tissues [19]. Indeed, after passing a pathological threshold, high ROS levels may lead to protein and DNA oxidation as well as lipid peroxidation, which can in turn affect the ETC and exacerbate ROS production. This may trigger a vicious cycle of oxidative stress that leads to cell death by apoptosis. In a previous review article, we discussed the effect of aging on the reduction/oxidation sate in the brain [1]. We emphasized that aging is associated with:

(i) A decrease in antioxidant defenses in the brain, namely a decrease in superoxide dismutase activity, in GSH content and GSH/GSSG ratio (reduced/oxidized glutathione).
(ii) An increase in oxidative damages, including increased free-radical level, DNA oxidative damages and lipid peroxidation.

Of note, neurons are particularly vulnerable to oxidative insults and mitochondrial dysfunction given that they are postmitotic and highly compartmentalized cells. Mitochondria are at the center of the free radical’s theory of aging by being a source and target of ROS.

## 3. Abnormal Tau and Bioenergetic Impairments

Disease-associated tau protein was shown to have a direct impact on mitochondrial function and to correlate with brain bioenergetic impairments. These points are discussed in the following sections.

### 3.1. Abnormal Tau and Mitochondrial Dysfunction

We recently reviewed the impact of abnormal tau on mitochondria [7]. Strikingly, abnormally hyperphosphorylated tau seems to impair almost every aspect of mitochondrial function, namely:

(i) Mitochondrial transport: abnormal tau inhibits anterograde transport of mitochondria along the axon, leading to a decreased number of mitochondria at the synapse, and mitochondrial perinuclear clustering [7,20].
(ii) Mitochondrial dynamics: abnormal tau induces mitochondrial network elongation by increasing mitofusin 2 levels (MFN2, involved in mitochondrial fusion) and by triggering dynamin-related protein 1 (DRP1, involved in mitochondrial fission) mislocalization and clustering in actin filaments [21,22].
(iii) Mitophagy: abnormal tau was shown to inhibit mitophagy by interacting with Parkin, a key protein involved in the mitochondria quality control process [23].
(iv) Mitochondrial bioenergetics: abnormal tau was shown to inhibit complex I activity, to decrease the mitochondrial membrane potential (proton-motive force necessary for ATP synthesis by OxPhos) and ATP levels, and to increase ROS production [24,25,26,27].

### 3.2. Abnormal Tau and Brain Glucose Metabolism

Increasing evidence shows that abnormal tau is associated with disturbances in brain glucose metabolism. In AD, decreased glucose uptake is an early event which starts in the hippocampal and entorhinal regions, as well as in the posterior temporal and parietal cortices [28,29]. Glucose hypometabolism then spreads to the dorsolateral prefrontal and premotor cortices, as well as in deeper brain regions, including the thalamus. Tau pathology seems to follow the same spreading pattern, starting in the trans-entorhinal regions (Braak stage I/II), then the limbic regions (Braak stage III/IV), and the neocortical regions (Braak stage V/VI) [30]. Of note, the link between tau pathology and impaired glucose metabolism is not specific to AD but can also be observed in other tauopathies, namely PSP and CBD, as similar brain regions present both tau deposition and glucose hypometabolism [31,32].

Using positron emission tomography (PET) to detect cerebral tau deposition (F-18 Tau-AD-ML 104) and glucose metabolism (F-18 FDG), Baghel and colleagues recently showed that tau pathology overlaps with glucose hypometabolism in the brain of AD patients, namely in the parietal lobe, temporal lobe, hippocampus, parahippocampus, frontal lobe, anterior and posterior cingulate, and precuneus [33]. In line with this, a significant negative correlation was observed between tau in the cerebrospinal fluid (CSF) and glucose uptake in the temporal, parietal and frontal region of the brain of AD patients [34]. In a multitracer PET study, Vlassenko et al. used different tracers to specifically measure aerobic glycolysis (AG), total glucose use (CMRGlc), and oxygen metabolism (CMRO_2_) in AD patients [35]. They showed a negative correlation between tau deposition and AG specifically in amyloid-β positive individuals, suggesting that the age-related decrease in brain glycolytic activity may accelerate tau pathology in patients with amyloid burden. 

Strikingly, both tau pathology and decreased glucose metabolism were shown to correlate with cognitive decline (Mini-Mental State Examination (MMSE) score) [28,29,33,36,37]. However, Chiotis and colleagues highlighted that the propagation of tau pathology (measure by tau-PET) is rather heterogenous among symptomatic AD patients, while changes in brain glucose metabolism is homogenous [38]. Therefore, glucose hypometabolism seems to be a better biomarker to track AD clinical progression. This seems to be more obvious in patients with mild cognitive impairments (MCI) where changes in glucose metabolism could predict a conversion to AD, while CSF biomarkers, including tau, could not [37]. Noteworthy, no correlation could be highlighted between amyloid-β pathology and cognitive impairments [39,40], or between amyloid-β pathology and glucose metabolism [34,38].

Further links between tau pathology and glucose metabolism were observed in pre-clinical AD [41]. In this recent study, regions with earlier tau spread were associated with stronger negative correlation with glucose metabolism in cognitively normal adults. Of note, in brain regions with low levels of tau, glucose hypermetabolism was observed. In MCI patients with low amyloid-PET, glucose hypermetabolism was associated with higher tau deposition and with worse cognitive performance [42]. One can hypothesize that this phenomenon is a compensatory mechanism aimed at counterbalancing the negative impact of abnormal tau on bioenergetics. This assumption is in line with the theory of the “Inverse Warburg effect on the origin of AD” proposed by Demetrius and colleagues, which asserts that the progression towards AD (sporadic form) involves three adaptive events: a hypermetabolic phase, a new metabolic equilibrium (prolonged prodromal phase), and a metabolic collapse [43,44]. More studies are required to better understand this phenomenon.

## 4. Age-Related Bioenergetic Impairments in the Brain: A Trigger for Tau Pathology?

Evidence has been linking disturbances in brain metabolism and tau pathology. For instance, studies have shown that cerebral hypoperfusion, which impairs brain energy metabolism, leads to an increase in tau abnormal phosphorylation in different animal models [45,46,47]. In the 3xTgAD model of AD, cerebral hypoperfusion (caused by unilateral common carotid artery occlusion) induced a significant increase in tau phosphorylation in the hippocampus of 3-month-old animals compared to controls (sham-operated mice) [45]. Moreover, cerebral hypoperfusion led to significant increase in phospho-tau levels not only in the hippocampus and cortex of 16-month-old 3xTgAD mice, but also in wild-type animals. No changes were detected regarding Aβ pathology. Similar observations were done in AD patients, in which cerebral hypoperfusion appeared to have a causative link with tau pathology but not Aβ pathology [47,48].

Intriguingly, brain aging is also marked by hypoperfusion due to the decrease in cerebral blood flow and the loss of BBB integrity [12,13,14], which may lead to deficits in nutrient import, including glucose, into the brain. As stated before, glucose hypometabolism appears to be a better biomarker to predict clinical progression of AD, compared to proteinopathy [36,37,38]. Nevertheless, there is a correlation between tau pathology and glucose hypometabolism in the brain of AD patients [33,34]. This might suggest that brain bioenergetic impairments are up-stream of tau deposition in AD. This point will be discussed in the next sub-sections.

### 4.1. Impaired Glucose Metabolism and Tau Pathology

The first evidence of the impact of glucose hypometabolism on tau pathology was provided by Lauretti and Praticò [49]. They showed that N2a mouse neuroblastoma cells cultured in glucose-free medium presented an increase in tau phosphorylation at epitopes Ser202/Thr205 and Ser404. As these epitopes correspond to the phosphorylation sites of the P38 mitogen-activated protein kinase (P38 MAPK), they demonstrated that glucose deprivation activates P38 MAPK, which phosphorylates tau protein. In addition, pharmacological inhibition of P38 MAPK prevented tau hyperphosphorylation and cell apoptosis. This interesting observations were later confirmed in vivo in mice expressing human tau (h-tau) [50]. Compared with the untreated group, h-tau mice treated with 2-deoxyglucose (2DG, inhibitor of glycolysis) presented an increase in tau phosphorylation, significant cognitive impairments, increased synaptic damage (decreased long-term potentiation) and increased apoptosis. Again, the abnormal tau hyperphosphorylation was due to the activation of the P38 MAPK pathway. 

**Figure 2:**
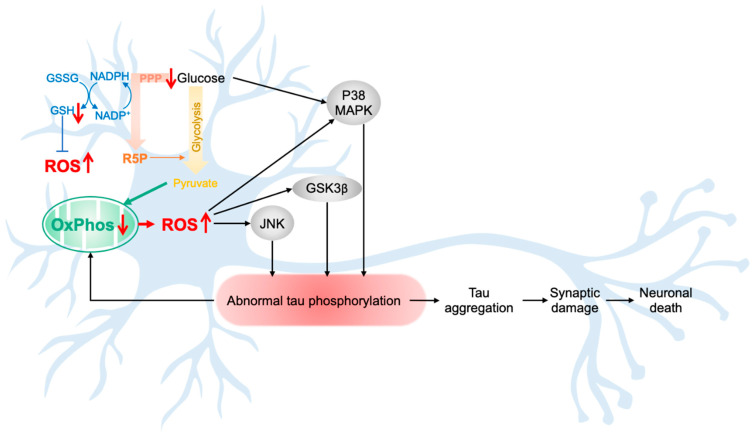
Potential impact of brain bioenergetic defects on tau pathology. Aging is associated with a decrease in brain glucose metabolism, impairments in mitochondrial function, namely decrease in mitochondrial oxidative phosphorylation activity (OxPhos) and increase in oxidative stress. Of note, the increase in reactive oxygen species (ROS) level in aging was linked to a decrease of brain antioxidant defenses and mitochondrial dysfunction, but might also be due to the decrease in glucose metabolism via the pentose phosphate pathway (PPP) in neurons, which is an important pathway involved in the generation of oxidized glutathione, a key regulator of reduction/oxidation state in neurons. The decrease in glucose/increase of ROS were shown to activate the tau-targeting kinases, P38 mitogen-activated protein kinase (P38 MAPK), glycogen synthase kinase-3β (GSK3β), and c-Jun N-terminal kinase (JNK), triggering abnormal tau hyperphosphorylation and aggregation. Abnormal tau was shown to induce synaptic damage and neuronal death, but also to impair mitochondrial function, which may exacerbate tau pathology. Abbreviations: GSH/GSSG, oxidized/reduced glutathione; NADP+/NADPH: oxidized/reduced nicotinamide adenine dinucleotide phosphate; R5P, ribulose-5-phosphate; red arrows: *↑* increase/*↓* decrease.

Taken together, these findings demonstrate that the decrease in glucose availability in the brain induces abnormal tau hyperphosphorylation, paralleled with synaptic failures and cognitive deficits (Figure 2).

### 4.2. Effects of Increased ROS Levels and Tau Pathology

Aging is associated with an increase of oxidative damage in the brain due to an increase in ROS production and/or decrease in antioxidant defenses [1]. Mounting evidence shows that increased ROS levels and oxidative stress negatively affect tau protein (reviewed in [51,52]).

For instance, human fibroblasts treated with hydrogen peroxide (H_2_O_2_) presented an increase in total tau levels, as well as an increase in tau phosphorylation at Ser396 and Ser202/thr205 that are AD-relevant tau phosphorylation sites [53].

Similarly, mice lacking the antioxidant enzyme SOD2 (superoxide dismutase 2 located in mitochondria), or SOD2 null mice, showed a significant increase in phospho-tau at Ser404, Ser396, Thr205 and Thr231 [54]. Abnormal tau hyperphosphorylation decreased after treatment with the antioxidant EUK189. These findings suggest that the increase in mitochondrial oxidative stress may be responsible for tau hyperphosphorylation. 

Interestingly, in a study conducted on mice bearing the P301S tau mutation, increases in oxidative stress and mitochondrial dysfunction (decrease in citrate synthase activity, manganese SOD activity, cytochrome c and cytochrome c oxidase) were detected before tau pathology, together with behavioral impairments [55]. This again suggest that oxidative stress and mitochondrial abnormalities appear prior to tau pathology.

The effects of oxidative stress on abnormal tau hyperphosphorylation seem to be linked to ROS-induced activation of kinases targeting tau [51,52]. Indeed, a chronic treatment of M17 human neuroblastoma cells with dl-buthionine-[S,R]-sulfoximine (BSO), an inhibitor of GSH synthesis, triggered oxidative stress in a time-dependent manner, paralleled with an increase of tau phosphorylation [56]. Among the pathways involved in tau hyperphosphorylation after chronic oxidative stress, a strong increase in the active form of the c-Jun N-terminal kinase (JNK) and p38 MAPK, as well as a decrease in the activity of protein phosphatase 2A (PP2A) was observed. In line with this, a significant increase in glycogen synthase kinase-3β (GSK3β) activity was measured in tau-transfected human embryonic kidney 293 (HEK293/Tau) cells treated with H_2_O_2_, together with tau hyperphosphorylation at Ser396, Ser404, and Thr231, three common GSK3β targeted sites [57]. 

JNK activation was also obvious in wild-type and mutant tau-expressing drosophila (UAS-tau^WT^ and UAS-tau^R406W^, respectively) compared to the native group [58]. In this model, inactivation of antioxidant pathways exacerbates tau-induced neuronal death, whereas up-regulation of antioxidant defenses suppressed tau-induced toxicity. Similarly, in P301S mice, oxidative stress was already detected at 7 months of age, probably inducing the increase in GSK3β level (detected at 10 months of age) and tau hyperphosphorylation in the cortex, hippocampus and spinal cord [55].

Taken together, these findings suggest that an increase in ROS levels lead to the activation of tau-phosphorylating enzymes, including p38 MAPK, JNK and GSK3β, resulting in tau abnormal hyperphosphorylation, synaptic failure and neuronal death (Figure 2).

## 5. Lifestyle Factors Influencing Brain Metabolism and Tau Pathology

It is becoming more and more obvious and accepted that lifestyle factors, including healthy diet, physical activity and cognitive stimulations, are able to substantially lower the risk of AD [59]. We will now discuss evidence showing that these lifestyle factors, which are also known to modulate brain bioenergetic metabolism, can influence tau pathology.

### 5.1. Diet

**Figure 3:**
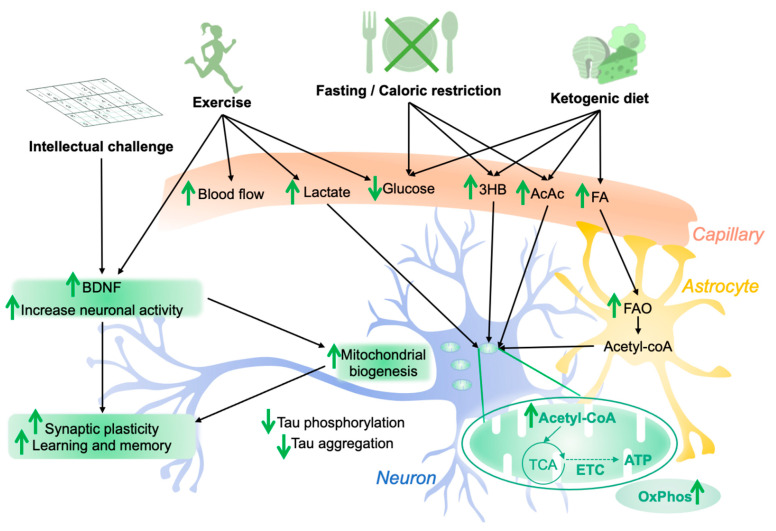
Influence of lifestyle factors on brain bioenergetics and tau pathology. Modified diet (e.g., fasting, caloric restriction, ketogenic diet) results in changes in brain levels of glucose, ketone bodies (3HB, AcAc) and fatty acids (FA), which are transported via the blood flow. FA can be metabolized by fatty acid oxidation (FAO) in astrocytes, providing energy substrates to neurons under the form of acetyl-coA. Ketone bodies can directly be used by neurons to fuel the tricyclic acid cycle (TCA) and oxidative phosphorylation (OxPhos), increasing mitochondrial bioenergetics and neuronal health. Physical exercise is known to improve brain function by increasing brain-derived neurotrophic factor (BDNF) levels, synaptic plasticity, mitochondrial biogenesis, and neurogenesis, improving cognitive functions such as learning and memory. Moreover, exercise is accompanied with an increase in lactate production by muscles. Lactate can be used by neurons as a bioenergetic substrate for OxPhos. Intellectual challenges were shown to increase neuronal network activity, increasing synaptic plasticity and cognitive function. These lifestyle factors were shown to decrease tau pathology, at least in in vivo animal models, but the underlying mechanisms, as well as their influence on tau pathology in humans, remain to be clearly identified. Abbreviations: 3HB, 3-β-hydroxybutyrate; AcAc, acetoacetate; ATP, adenosine triphosphate; ETC, electron transport chain; green arrows: *↑* increase/*↓* decrease.

Dietary energy restriction, achieved by caloric restriction (CR) or intermittent fasting (IF), is a way to force the liver to use fatty acids from adipose tissues instead of glucose. The liver then produces ketone bodies (e.g., acetoacetate: AcAc; 3-β-hydroxybutyrate: 3HB) that are released in the blood flow and can be used as energetic substrates by the brain (Figure 3) [2]. Of note, fatty acids constitute an important energy source for cells, and can be metabolized by astrocytes. Recent works have suggested that fatty acid β-oxidation (FAO), which takes place within mitochondria, may constitute an alternative pathway providing bioenergetic substrates in the brain, [60]. CR, IF and ketogenic diet (KD) were shown to promote brain health [2,61]. 

The influence of diet on tau pathology was demonstrated in in vivo studies using different animal models of tauopathies (Table 1). Indeed, CF and IF attenuated cognitive deficits in triple transgenic AD 3xTgAD mice [62]. However, only CR decreased tau phosphorylation in the hippocampus. Therefore, the authors suggested that CR and IF improved age-related cognitive function by other mechanisms that remain to be determined. In the same model, mice fed with a ketone ester-based diet showed an improvement in cognitive function when compared to mice fed with the carbohydrate-enriched (CHO) control diet [63]. In addition, tau abnormal hyperphosphorylation was significantly reduced in the hippocampus, cortex, and amygdala. In Tg4510 mice, no effect of CR or KD was observed on tau pathology, while only a slight improvement of cognitive function was detected [64,65].

Chronic traumatic encephalopathy (CTE) caused by repetitive traumatic brain injuries (TBI) is characterized by the presence of hyperphosphorylated tau in the brain [77]. In a recent study, h-tau mice were fed with so-called NutriFusion diets, which consist of a normal diet supplemented with a combination of vitamins, fruit and vegetables, for 2 months prior to the induction of repetitive mild TBI [66]. NutriFusion diets prevented tau hyperphosphorylation and improved learning and memory after TBI, when compared to animals fed with the normal diet alone. This study demonstrates that consumption of nutrient-rich diets directly influences the pathological outcomes after TBI, including tau pathology. 

Metabolic disorders, such as obesity and type 2 diabetes mellitus (T2DM), are considerate as risk factors for AD. Intriguingly, in obese h-tau mice, CR was shown to increase tau aggregation [68]. Similarly, in a model of diabetic rats, an increase of tau phosphorylation was observed in the CR group compared to animals fed *ad libitum* [78]. These studies suggest that strategies aimed at reducing metabolic disorders and AD risk must be selected carefully to avoid exacerbation of pathologies.

In humans, KD showed beneficial effects on cognition in MCI and AD patients (reviewed in [79]). However, studies investigating the effects of diet on tau pathology using large sample sizes and longitudinal data are still missing. Nevertheless, Neth and colleagues recently assessed the effects of a modified Mediterranean-ketogenic diet (MMKD, which induces ketosis) and the American Heart Association Diet (AHAD, a low fat diet) on CSF AD biomarkers and cognition in adults with subjective memory complaints (SMC) and MCI [80]. They showed that after 6 weeks of modified diet (MMKD or AHAD) followed by 6 weeks wash out (normal diet), CSF levels of tau decreased in MCI patients. In addition, brain ketone uptake increased after MMKD, suggesting that ketogenic dietary intervention promotes cerebral ketosis. However, although MMKD was associated with better performance on the Free and Cued Selective Reminding Test (FCSTR), no effects were observed on the ADAS-Cog12, a test of general cognition that includes items related to executive function, attention, verbal abilities, and memory. The authors suggested that 6 weeks of MMKD may have been an insufficient treatment length to clearly impact cognition. Further studies with longer diet duration, and larger sample sizes are now needed.

### 5.2. Physical Exercice

Regular aerobic exercise was shown to improve brain function, including attention, memory, and learning [2]. These effects may be explained by the fact that physical exercise increases (among others):

(i) Blood flow, which improves nutrient import into the brain (Figure 3).
(ii) Lactate levels in the blood due to muscular activity. Lactate can then be transported into the brain and directly used by neurons as a bioenergetic substrate for the TCA cycle and OxPhos.
(iii) Brain-derived neurotrophic factor (BDNF) levels, and other proteins involved in mitochondrial biogenesis, which increases synaptic plasticity, learning and memory, as well as improves resistance to stress and neurodegeneration (reviewed in [2]).

Beneficial effects of physical exercise on tau pathology were shown in different animal models (Table 1). For instance, in the THY-Tau22 transgenic model of AD-like tau pathology, 9 months voluntary physical exercise (running wheel) starting at 3 months of age was able to prevent tau-induced memory deficits, and decreased abnormal tau phosphorylation in the hippocampus [69]. Similarly, 12-weeks of forced treadmill exercise significantly reduced total tau and hyperphosphorylated tau levels in the spinal cord and hippocampus of P301S mice, together with improvements in locomotor and exploratory activity when compared to sedentary animals [67]. These effects were not coupled with a decrease of neuronal death in the hippocampus, which suggests that a tau-independent mechanism may be at play with regards to the improvement of behavioral defects. More recently, Liu and colleagues showed that a short-term resistance training protocol, in which mice have to climb up a 1-m ladder with a progressively heavier weight loading, decreased total and phospho-tau levels in the cortex and hippocampus of Tg3xAD mice, compared to sedentary mice [72]. In addition, an improvement in cognitive performance was observed, together with an increase in synaptic plasticity, and activation of the JNK pathway. In line with this, in a streptozotocin (STZ)-induced sporadic AD rat model, 4-weeks swimming exercise prior to STZ injection was able to decrease tau hyperphosphorylation and oxidative damages, and to prevent STZ-induced neurodegeneration and cognitive dysfunction [70].

As mentioned before, obesity is considered to be a risk factor for AD. In obese h-tau mice, voluntary exercise reduced tau phosphorylation while CR increased tau aggregation [68]. Similarly, wild-type rats fed with high-fat diet for 20 weeks showed a significant increase of abnormal tau phosphorylation in the cortex and hippocampus [71]. After 8 weeks treadmill exercise, obese rats presented a decrease in tau hyperphosphorylation and aggregation, together with improved learning and memory performance, compared to control rats (no exercise).

Although animal studies indicate that physical exercise has beneficial effect against tau pathology, human studies provide less compelling evidence. It appears that physical exercise, especially high intensity physical exercise, lowers the levels of CSF tau and PET-quantified tau in cognitively normal individuals (reviewed in [81]). In addition, increased length and/or intensity of physical exercise seem to be more efficient in reducing tau levels [81,82]. However, these effects were not observed in MCI and AD patients. Recently, Frederiksen and colleagues carried out a systematic review of the observational studies of physical activity and AD biomarkers in healthy subjects, SMC, MCI and AD patients [83]. They show that most of the studies do not find a significant effect of physical exercise on AD biomarkers. Nevertheless, they emphasize that only a few longitudinal studies were performed, with small sample sizes, which may limit the conclusions. As evidence from animal models suggests that physical exercise is more beneficial within pre-symptomatic stages of AD [81], large randomized controlled trials in humans are warranted, especially at very early disease stages.

### 5.3. Intellectual Challenge

Intellectual challenges, such as cross-word puzzles or sudoku, were shown to increase neuronal network activity, which leads to the up-regulation of BDNF levels (reviewed in [2]). Both BDNF and neuronal activity regulates signaling pathways leading to an increase in mitochondrial biogenesis, synaptic plasticity, and learning and memory. Therefore, intellectual stimulation in brain aging might be a way to, at least, slow down cognitive decline in elderly subjects.

Intellectual stimulation was shown to have beneficial effects on tau pathology in different animal models (Table 1). In fact, in animal studies, effects of intellectual stimulation are assessed by placing the animals in an enriched environment (EE), usually consisting of enlarged cages where various objects are placed, added, and exchanged, every week for several weeks. In different AD and tau transgenic mouse models, EE was shown to decrease tau pathology, increase BDNF levels, increase long-term potentiation, as well as to enhance neurogenesis [73,74,75]. Similarly, obese rats (fed with high-fat or high-sucrose diets) housed in EE showed similar tau levels than control rats (fed with standard diet), while rats housed in a normal environment presented significantly increased tau levels [76].

As was also the case with dietary intervention and physical exercise, studies assessing the effects of cognitive stimulation on tau pathology in humans are scarce. Even though there is some evidence showing that cognitive stimulation improves cognitive function in AD patients [84,85], the effects on tau pathology were, to our knowledge, not investigated.

Taken together, most animal studies suggest that modified diet (CR, IF, KD), increased physical exercises, and cognitive stimulation, decrease tau pathology and improve cognitive function. Human data seem to be inconclusive because only a few longitudinal studies assessing the level of tau pathology were performed, and usually with small sample size. Nevertheless, Dhana and colleagues recently combined data from two longitudinal studies (total: 2765 participants, about 6 years follow-up) which defined a healthy lifestyle score on the basis of: healthy diet (high-quality Mediterranean-DASH Diet Intervention for Neurodegenerative Delay diet), moderate/vigorous-intensity physical activity (>150 min/week), engagement in late-life cognitive activities, as well as non-smoking and light to moderate alcohol consumption [59]. They clearly showed that the risk of AD dementia was 37% lower in individuals combining two or three of the above-mentioned healthy lifestyle factors, and up to 60% lower in those combining four of five of these factors. More studies are now required to understand the underlying mechanisms, especially regarding the effects on brain metabolism and tau pathology.

## 6. Conclusions

In summary, aging and tau protein induce the same bioenergetic impairments in the brain, namely mitochondrial dysfunction and increased oxidative damage. In addition, age-related defects in brain glucose metabolism seem to correlate with the progression of tau pathology and cognitive impairments in AD. Of note, brain regions that are affected by abnormal tau in PSP and CBD also present glucose hypometabolism [31,32], suggesting that the link between tau pathology and impaired glucose metabolism is not specific to AD but is also observed in other tauopathies.

**Figure 4:**
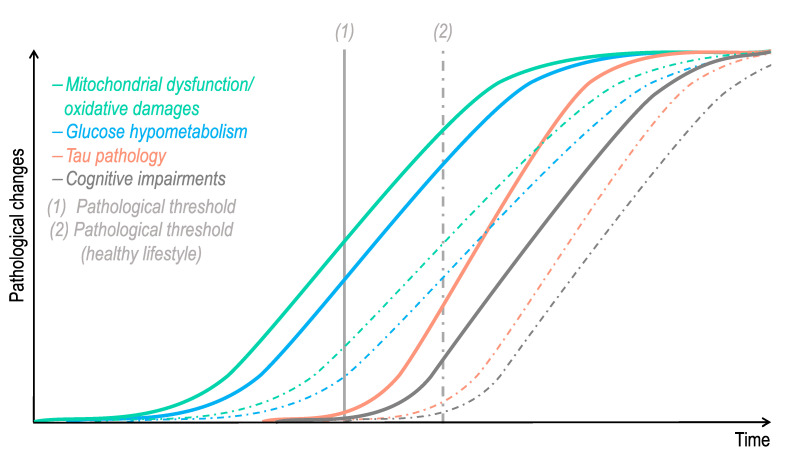
Hypothetical time course of pathological changes in Alzheimer’s disease-related tauopathy. Impairments in mitochondrial function, oxidative stress, and glucose hypometabolism are features that are observed in “normal” aging. One can hypothesize that after passing a pathological threshold (1), which differs according to each individual lifestyle/genetic background, these age-related bioenergetic defects trigger tau pathology. In turn, abnormal tau exacerbates brain bioenergetic dysfunctions, precipitating cognitive impairments and dementia. The dotted lines represent pathological changes that would be delayed in an individual with a healthy lifestyle, combining, among others, a healthy diet, physical activity, and cognitive stimulation.

Mounting evidence showed that alterations in brain glucose metabolism, as well as an increase in ROS levels, may activate tau-targeting kinases, triggering an aberrant tau hyperphosphorylation. Therefore, one can hypothesize that after passing a pathological threshold, the age-related bioenergetic impairments observed in the brain (decreased glucose metabolism/increase oxidative damages) lead to tau abnormal phosphorylation and aggregation into NFTs (Figure 4). In turn, abnormal tau impairs mitochondrial function, exacerbating biogenetic defects, as well as its own phosphorylation state (see also Figure 2). 

Although more longitudinal studies are required to clearly assess the effects of diet, physical activity and cognitive stimulation, on tau pathology, it seems that these lifestyle factors, and especially their combination, significantly lower the risk of AD [59]. Additionally, it appears that high-educated AD patients are able to tolerate more tau pathology than low-educated AD patients with comparable cognitive impairments [86]. This supports the idea that cognitive reserve can preserve brain functionality despite the accumulation of pathology. Thus, lifestyle may determine the individual pathological threshold after which age-related brain bioenergetic impairments will trigger tau pathology and cognitive dysfunction. 

## Figures and Tables

**Table 1:** Effects of modified diet, physical exercise and cognitive stimulation on tau pathology in animal models.

Model	Methods	Main Effects of Modified Diet/Physical Exercise/Cognitive Stimulation	Ref.
3xTgAD mice	Calorie restriction (CR) and intermittent fasting (IF) beginning at 3 months of age	Suppression of tau pathology with CR, but not IF, in the hippocampus of old miceCR and IF ameliorate age-related cognitive deficits	[62]
Tg4510 mice	12-weeks caloric restriction (CR)	No effet of CR on tau pathologyImprovment of short-term memory and contextual memory (trend), but not spatial memory	[64]
3xTgAD mice	8-months ketone ester-based diet	Reduced levels of hyperphosphorylated tau deposition in the CA1 and CA3 regions of the hippocampus, amygdala, and cortexSlight improvement of congnitive function (learning and memory)	[63]
Tg4510 mice	3-months ketogenic diet (KD)	Enhancement of motor performanceNo effect of KD on cognitive function and tau pathology	[65]
h-tau mice with repetitive mild traumatic brain injuries (rmTBI = controlled, repetitive closed head impacts)	NutriFusion diets * for 2 months prior to the rmTBI	Prevention of tau pathologyImproved behavioral outcomes after rmTBI, including learning and memory	[66]
P301S tau mice	12-weeks of forced treadmill exercise	Significant reduction in full-length and hyperphosphorylated tau (spinal cord and hippocampus)Reductin of insoluble tau in the spinal cordImprovment of locomotor and exploratory activityNo significant attenuation of neuronal death in the hippocampus	[67]
h-tau mice	2-months voluntary physical exercise (running wheel) and caloric restriction in h-tau mice under high caloric diet (obese mice)	Reduction of tau phosphorylation with physical activityIncreased tau aggregation with caloric restriction in the brain of obese mice	[68]
THY-Tau22 mice	9-months voluntary physical exercise (running wheel)	Prevention of memory deficits by physical exerciseDecreased tau pathology in the hippocampus	[69]
streptozotocin (STZ)-induced sporadic AD rats	Swimming exercise training for 4 weeks before STZ injection	Decrease of STZ-induced tau hyperphosphorylation and oxidative damagesPrevention of STZ-induced cognitive dysfunction and synaptic loss/cell death in the hippocampal CA1 region	[70]
Obese Sprague-Dawley rats (high-fat diet (HFD) for 20 weeks)	8-weeks treadmill exercise (progressively increasing load intensity)	Decreased hyperphosphorylation and aggregation of Tau proteinImprovment of cognitive function (learning and memory)	[71]
3xTgAD mice	Short-term resistance training (climbing up a 1-m ladder with a progressively heavier weight loading)	Decreased total and hyperphosphorylated tau in the frontal cortex and hippocampusImprovement of cognitive performance	[72]
APPswe/PS1ΔE9 mice	3 h/day environmental enrichment (EE) for 1 or 2 months = mice transferred in enlarged cages with running wheels, colored tunnels, visual stimulating toys. Objects in the cage were repositioned for novel stimulation every day	Enhancement of neurogenesisSignificant reduction in levels of hyperphosphorylated tau in the hippocampus and cortex Enhancement of hippocampal long-term potentiation	[73]
5xFAD mice	8 weeks in EE conditions = cages in which plastic tubes, plastic dolls or toys were added, extracted, or changed every week	Reduced cognitive deficitsIncreased neuroplasticityDecrease of tau phosphorylation	[74]
E257K/P301S-Tau (DM-Tau Tg) mice	Mice housed for 9 months in EE versus regular environment cages.EE = every week, mice are transferred to new enlarged cages equipped with a running whell and differently shaped objects (tunnels, boxes, cubes, balls, ladder, labyrinth)	Reduced neurofibrillary tangle (NFT) burdenIncreased neurogenesisIncrease in brain-derived neurotrophic factor (BDNF) levelsTrend toward improvement in cognitive tasks	[75]
High-fat, high-sucrose fed rats	Rats housed in EE = cages containing objects such as toys, tunnels, running wheels, stairs and platforms. The EE design was changed twice a week	Normalization of tau protein level to the control group (normal diet)	[76]

* NutriFusion diets = normal diet with ~ 2% supplementation of the different materials NF-216 (GrandFusion—Fruit and Veggie ^#^1 Blend), NF-316 (GrandFusion—Fruit ^#^2 Blend), and NF-416 (GrandFusion—Vegetable ^#^3 Blend).
